# Convergence of YAP/TAZ, TEAD and TP63 activity is associated with bronchial premalignant severity and progression

**DOI:** 10.1186/s13046-023-02674-5

**Published:** 2023-05-08

**Authors:** Boting Ning, Andrew M. Tilston-Lunel, Justice Simonetti, Julia Hicks-Berthet, Adeline Matschulat, Roxana Pfefferkorn, Avrum Spira, Matthew Edwards, Sarah Mazzilli, Marc E. Lenburg, Jennifer E. Beane, Xaralabos Varelas

**Affiliations:** 1 Department of Medicine, Computational Biomedicine Section, Boston University Chobanian & Avedisian School of Medicine, 72 East Concord Street, Boston, MA 02118 USA; 2 Bioinformatics Program, Boston University, 72 East Concord Street, Boston, MA 02215 USA; 3 Department of Biochemistry and Cell Biology, Boston University Chobanian & Avedisian School of Medicine, 72 East Concord Street, Room K620, Boston, MA 02118 USA; 4 Johnson and Johnson Innovation, Cambridge, MA 02142 USA

**Keywords:** Bronchial premalignant lesions, YAP, TAZ, TEAD, TP63, Immune evasion, CIITA, MHCII

## Abstract

**Background:**

Bronchial premalignant lesions (PMLs) are composed primarily of cells resembling basal epithelial cells of the airways, which through poorly understood mechanisms have the potential to progress to lung squamous cell carcinoma (LUSC). Despite ongoing efforts that have mapped gene expression and cell diversity across bronchial PML pathologies, signaling and transcriptional events driving malignancy are poorly understood. Evidence has suggested key roles for the Hippo pathway effectors YAP and TAZ and associated TEAD and TP63 transcription factor families in bronchial basal cell biology and LUSC. In this study we examine the functional association of YAP/TAZ, TEADs and TP63 in bronchial epithelial cells and PMLs.

**Methods:**

We performed RNA-seq in primary human bronchial epithelial cells following small interfering RNA (siRNA)-mediated depletion of YAP/TAZ, TEADs or TP63, and combined these data with ChIP-seq analysis of these factors. Directly activated or repressed genes were identified and overlapping genes were profiled across gene expression data obtained from progressive or regressive human PMLs and across lung single cell RNA-seq data sets.

**Results:**

Analysis of genes regulated by YAP/TAZ, TEADs, and TP63 in human bronchial epithelial cells revealed a converged transcriptional network that is strongly associated with the pathological progression of bronchial PMLs. Our observations suggest that YAP/TAZ-TEAD-TP63 associate to cooperatively promote basal epithelial cell proliferation and repress signals associated with interferon responses and immune cell communication. Directly repressed targets we identified include the MHC Class II transactivator CIITA, which is repressed in progressive PMLs and associates with adaptive immune responses in the lung. Our findings provide molecular insight into the control of gene expression events driving PML progression, including those contributing to immune evasion, offering potential new avenues for lung cancer interception.

**Conclusions:**

Our study identifies important gene regulatory functions for YAP/TAZ-TEAD-TP63 in the early stages of lung cancer development, which notably includes immune-suppressive roles, and suggest that an assessment of the activity of this transcriptional complex may offer a means to identify immune evasive bronchial PMLs and serve as a potential therapeutic target.

**Supplementary Information:**

The online version contains supplementary material available at 10.1186/s13046-023-02674-5.

## Background

Lung cancer accounts for the largest number of deaths in the United States among all cancer types, making up over 20% of cancer-related deaths in 2020 [1]. The development of lung squamous cell carcinoma (LUSC), one of the most common subtypes of lung cancer, is preceded by the formation of bronchial premalignant lesions (PMLs) that are characterized by the abnormal expansion and morphological alteration of airway basal cells [2] that progress through a series of histological grades, from normal, hyperplasia, metaplasia to dysplasia. Our poor understanding of the early molecular events associated with these precancer states makes it difficult to develop potential interception strategies for LUSC [3, 4]. Previous studies profiling gene expression in bronchial PML samples have suggested that progressive higher-grade lesions show immune evasion profiles, including impaired antigen presentation and decreased lymphoid and myeloid populations [5–9]. Although immune evasion is a feature of LUSC, the mechanisms contributing to similar phenotypes in bronchial PML progression is poorly understood.

A transcription factor important for controlling bronchial basal cell identity is the p53 family member TP63 (also known as p63) [10, 11], which is encoded by the *TP63* gene and is expressed as several isoforms [12]. Amplification or overexpression of *TP63* are frequently observed in squamous cell carcinoma, including LUSC [13, 14], and ectopic expression of the ΔNp63 isoform (ΔNp63) has been shown to drive to the development of squamous metaplasia in the mouse lung and promote proliferative phenotypes in skin epithelial basal cells [15, 16]. The activity of TP63 is regulated by several transcriptional co-factors, including the Hippo signaling pathway effectors YAP and TAZ in a context-specific manner. For example, YAP has been shown to bind and stabilize ΔNp63 in human keratinocyte, lung cancer, and head and neck squamous cell lines [17, 18], and association between YAP and ΔNp63 has been suggested to regulate the growth of basal epithelial cells of the mouse trachea [19]. YAP binding to TP63 results in strong transcriptional co-activation [18, 20], suggesting a cooperative relationship between these factors. However, these interactions seem to be complex as they may regulate apoptosis [20] or cell growth depending on cellular context [19]. TP63 activity has been shown to promote nuclear YAP activity in head and neck squamous cell carcinoma cells [21], but interestingly YAP has been suggested to repress the ΔNp63 expression in breast and lung cancer cell lines [22, 23]. Thus, there appears to be a multifaceted relationship between these factors in the context of stem cells and cancer, which we are only starting to understand.

Recent evidence has implicated the aberrant activity of YAP/TAZ in bronchial PML development. For example, YAP/TAZ regulated transcription is associated with the progression of human PMLs, and the aberrant activation of YAP/TAZ in the bronchial epithelium of mice drives epithelial growth and PML-like pathology [24]. YAP and TAZ encoding genes (*YAP1* and *WWTR1*, respectively) are frequently amplified in squamous carcinoma [25] and ample evidence has suggested the oncogenic role of YAP/TAZ across multiple cancer types [26, 27]. YAP and TAZ have also emerged as key transducers of pro-oncogenic mechanical signals, such as those induced by changes in microenvironment elasticity [28, 29]. YAP/TAZ functions rely on their ability to associate with the TEAD family of transcription factors [30, 31], including the essential roles for YAP/TAZ in the development and homeostasis of the lung [19, 32, 33].

The functional relationship between YAP/TAZ, TEAD and TP63 has been poorly explored in the lung. Given the implication of these factors in bronchial PML development, we set out to investigate the binding pattern and gene expression program of these factors in human bronchial epithelial cells (HBECs) proliferating in a basal stem cell state. We found that YAP, TEADs, and TP63 associate with shared chromatin regions and co-regulate a gene expression program in proliferating HBECs. Integrating genomic binding and gene expression profiling, we demonstrate that gene targets directly induced by TEAD and TP63 are enriched for pro-proliferative genes and those directly repressed are enriched for genes involved in interferon responses and immune regulation. Genes repressed by YAP/TAZ-TEAD-TP63 are notably enriched among the genes down-regulated in progressive/persistent PMLs and includes *CIITA* (also known as MHC2TA), known as a “master” transcriptional co-activator of major histocompatibility complex (MHC) class II gene transcription. Our data suggest that a YAP/TAZ-TEAD-p63 regulated network contributes to a bronchial basal cell proliferative state and the immune-evasive microenvironment observed in lung PMLs. Taken together, our results provide insight into the functions of transcriptional complexes that contribute to the early stages of lung carcinogenesis and offer potential new avenues to develop lung cancer interception strategies.

## Methods

### Primary human bronchial epithelial cell culture

Primary HBECs were cultured in PneumaCult EX Plus media (StemCell Technologies). Information about the individual patient HBECs used in the study is outlined in Supplementary Table S1. siRNA transfection was carried out with Lipofectamine RNAimax (Invitrogen, 13,778,150) on low density proliferating cells and were maintained in submerged culture for 48 h before lysis. siRNA information used in the study are listed in Supplementary Table S2.

### Immunoprecipitation and immunoblotting

HBECS were cultured in PneumaCult EX plus (StemCell Technologies) and proliferating cells were lysed in Tris-buffered saline with 0.1% Tween (TBS-T) detergent. Lysates were subjected to immunoprecipitation using an anti-pan-TEAD antibody to isolate endogenous TEAD proteins and then analyzed by immunoblotting using an anti-TP63 antibody.

### Proximity ligation assays

Primary HBECs were seeded in 8 well chamber slides at density 5000 cells per well. Cells were fixed in 4% PFA in PBS (pH-7.4) for 10 min and Proximity ligation assays (PLA) were performed using Duolink In Situ Detection Reagents FarRed (DUO92013, Sigma-Aldrich) according to the manufacturer instructions. Primary antibodies used included anti-P63 (1:1000, CM163B, Biocare Medical), anti-YAP (1:500, 14074S, CST), anti-YAP (1: 200, 12395S, CST) and anti-pan-TEAD (1:150, 13295S, CST). Slides were mounted using ProLong Gold Antifade Mountant with DAPI (P36941, Invitrogen). Imaging (z-stacks – 19uM) was carried on a LSM880 AiryScan in super resolution mode (Plan-Apochromat 40x/1.3 Oil DIC M27 Objective, MBS 488/561/633). Images of 5 fields of view (FOV) with ~ 7–20 cells were captured per condition and quantified using previously described pipelines on Image J [34] (minimum of 45 cells quantified per condition). Data was analyzed using Graphpad Prism (9.0.2).

### TP63 and isoform expression data analysis in TCGA LUSC and PML data

Copy number alteration data (from GISTIC) for The Cancer Genome Atlas (TCGA) LUSC samples (PanCancer Atlas; *N* = 487), TP63 amplification frequency and expression level z-score relative to normal samples across TCGA cancer types were downloaded from cBioPortal [35–37]. A list of transcription factor genes in LUSC was obtained from AnimalTFDB 3.0 [38]. TCGA LUSC gene and transcript level expression data of the TCGA LUSC samples were downloaded using TCGAbiolinks [39] (legacy data) for primary tumor (*N* = 501) and normal tissue (*N* = 51) samples. The count data were normalized using the trimmed mean of M-values (TMM) from edgeR R package [40] and Voom-transformed [41]. For analysis on TAp63 and ΔNp63, raw counts related to each isoform were summed before normalization based on annotation from UCSC and Ensemble genome browser (excluding retained introns). Gene and TP63 isoform over-expressions were examined with a linear model comparing tumor to normal samples adjusting for the plate. To assess the association between TP63 isoform expression level and the histological grades in Beane et al. [5], same normalization was performed, and a linear mixed-effect model was fitted with the lesion grade as the main independent variable, adjusting for sequencing batch and median TIN and the patient was adjusted as a random effect.

### RNA-seq experiments

For generating the YAP/TAZ, TEAD and TP63 regulated gene expression signature in human airway cells, HBECs (Supplementary Table S1) were cultured in PneumaCult EX plus and transfected with control siRNA (three distinct siRNA controls) or siRNA targeting Yap/Taz TEAD 1-4 or TP63 (Supplementary Table S2) and after 48 hours of growth cells were lysed and RNA was extracted from triplicate samples for RNA-sequencing. Sequencing libraries were prepared using Illumina TruSeq RNA Sample Preparation Kit v2 and sequenced on the Illumina HiSeq 2500 platform to generate single end 76-nt reads.

FASTQ files were demultiplexed and created by Illumina BaseSpace. The quality of the FASTQ files was examined with FastQC [42]. The samples were aligned to the build version hg19 of the human genome using STAR 2-pass alignment [43]. RSEM [44] was then used the quantify the gene and transcript counts using Ensembl v75 annotation, and RSeQC [45] was used to calculate the quality metrics. The count data were normalized by the library sizes using the TMM and transformed into log2 counts per million using edgeR R package [40].

### ChIP-seq and ChIP-qPCR experiments

HBECs (Supplementary Table S1) for ChIP were cultured in PneumaCult EX Plus media and cross-linked in 1 mM EGS in PBS for 30 min followed by a 1% formaldehyde treatment for 10 min. Fixation was subsequently neutralized with 0.125 M glycine in PBS. Harvested chromatin was isolated as single samples from each patient line, sonicated using the Bioruptor UCD- 200 and the incubated with the following antibodies at 4 °C overnight: Rabbit anti-Yap (Abcam, Cat# ab52771, 3ug), Rabbit anti-TEAD (AvivaSysBio, Cat# ARP38276, 1ug), and Mouse anti-P63 (Biocare # CM163, 5ug). Immunoprecipitated complexes were collected by Protein A/G Magnetic beads (Pierce, 8802). Samples were washed with low salt buffer (20 mM Tris, 140 mM NaCl, 1 mM EDTA, 0.1% NaDeoxycholate, 0.1% SDS, 1% Triton X-100), followed by a high salt buffer (20 mM Tris, 500 mM NaCl, 1 mM EDTA, 0.5% NaDeoxycholate, 1% Triton X-100), and a LiCl buffer (20 mM Tris, 1 mM EDTA, 0.1% NaDeoxycholate, 1% Triton X-100, 250 mM LiCl). Chromatin was de-crosslinked overnight at 65C and purified using the Qiaquick PCR purification kit (Qiagen, 28,104).

For ChIP-seq, the purified DNA was ligated to specific adaptors and sequenced using DNB-seq, performed by BGI, to a depth of 40 million reads. The ChIP-seq fastq files were aligned to the build version hg19 of the human genome using Bowtie2 [46] with the default parameters. Reads that were unmapped, not primary alignment or with MAPQ score lower than 30 were removed. Duplicated reads were marked by Picard [47] and were discarded from the alignments and the resulting SAM files were converted to BAM format with samtools [48]. Peak-calling was performed for each individual replicate against the IgG control ChIP-seq consistently using the narrow-peak mode from Model-based Analysis for ChIP-Seq (MACS2) [49] at a p-value cutoff of 0.05 with nomodel option and extsize of 150. Additionally, peaks were filter by summit fold-change > 2. Peaks within the blacklisted regions (hgdownload.cse.ucsc.edu/goldenpath/hg19/encodeDCC/) were removed. Overlapped peaks between replicates were then identified using the findOverlapsOfPeaks function from the ChIPpeakAnno R package [50] and were used for downstream analysis. Overlapped peaks between ChIP-seq experiments were found using the findOverlapsOfPeaks function from the same package.

The normalized read density for each factor was calculated using callpeak function of MACS2 (-B –SPMR –nomodel –extsize 150) from pooled replicates for genome track visualization using karyoploter R package [51] and read coverage visualization within up- and down-stream 2 kb window around the peak center. Significance of peak overlap was calculated with the enrichPeakOverlap function from ChIPseeker R package [52]. Motif enrichment analysis was done within the YAP peak, YAP-TEAD overlapped peak, and the YAP-TP63 overlapped peak regions using findMotifsGenome.pl function from HOMER software suite [53] with the default parameters. The distances between peak locations and TSS for each factor were calculated using the annotatePeakInBatch function from the ChIPpeakAnno R package [50] and EnsDb.Hsapiens.v75. Peak distributions by the genomic elements were done with ChIPseeker [52].

For ChIP-qPCR, purified DNA isolated from ChIP reactions with Rabbit anti-Yap (Abcam, Cat# ab52771), Rabbit anti-TEAD (AvivaSysBio, Cat# ARP38276), or Mouse anti-P63 (Biocare # CM163) was used in qPCR reactions to assess predicted binding sites based on ChIP-seq analysis. Specifically, binding sites upstream of the *CIITA* gene (*chr16:10,943,592–10,943,925)* were examined using the primers listed in Supplementary Table S3. Three independent experiments were analyzed relative to the input and profiled statistically using Graphpad Prism (9.0.2).

### Derivation of gene expression signature from RNA-seq siRNA experiments

Gene expression signatures were generated comparing each siRNA knockdown with the control experiments in HBECs separately. First, we excluded genes from the count table if the interquartile range was equal to zero or the sum of counts was less or equal to 1 across samples. This yielded 13,976, 13,918, 13,938, and 13,859 genes for the siYT, siTEAD, siTP63, and siLATS experiments, respectively. The remaining genes were TMM normalized again. Then, the data was Voom-transformed and the differentially expressed genes associated with siRNA treatment were identified using a linear model in limma R package with treatment as the main independent variable, adjusting for cell line [41, 54]. Genes significantly associated with siRNA treatment were filtered at False Discovery Rate (FDR) < 0.05 and absolute log fold change greater than 0.5. Genes were ranked by the t-statistic for their association with treatment effect to generate the rank list for each siRNA. The enrichment of differentially expressed genes on rank lists from another siRNA KO experiment was examined by Gene Set Enrichment Analysis (GSEA) [55] using the fgsea R package [56]. Expression residual values adjusting for cell line were used for heatmap visualization using the ComplexHeatmap R package [57].

### Derivation of direct target genes of TEAD and TP63 from ChIP-seq experiments

Genes with a transcriptional start site (TSS) within 50 kb from TEAD-TP63 overlapped peaks were assigned as direct target genes. To account for the potential long-range interaction, we utilized promoter capture Hi-C interaction data of lung tissue from 3div.kr [58]. P-value cutoff of 0.05 was used to filter the promoter-promoter and promoter-other interactions, which resulted in 15,545 and 52,254 pairs of chromatin interactions, respectively. Genes with TSS overlapping with a promoter-containing fragment that had interacting fragment overlapped with a TEAD-TP63 overlapped peak were assigned as direct target gene. Then, the target gene sets were filtered based on their association with siYT, siTEAD and siTP63 treatments. Genes significantly up-regulated (FDR < 0.05 and log fold-change > 0.5) in all siRNA treatments compared to the controls were assigned as “repressed targets”, whereas genes significantly down-regulated (FDR < 0.05 and log fold-change < -0.5) were assigned as “induced targets.” Functional pathway enrichment analysis for the TEAD-TP63 induced and repressed target genes were performed using the hypergeometric test implemented in the R package hypeR [59] and the Molecular Signatures Database (MSigDB) from the Broad Institute. The enrichment of TEAD-TP63 induced and repressed target genes within PML co-expressed gene modules were examined with Fisher’s exact test.

### Computational analyses of TEAD-TP63 direct target genes in human patient data

We obtained bulk gene expression profiles of endobronchial biopsies spanning various PML histological grades and progression status from two studies: the discovery and validation cohort from Beane et al. (GSE109743; discovery cohort with 190 biopsies from 29 subjects; validation cohort with 105 biopsies from 20 subjects) [5] and Merrick et al. (GSE114489; 63 biopsies from 42 subjects) [9]. For the samples from Beane et al., the residual expression values adjusting for batch and RNA quality measured by the transcript integrity number (TIN) [45] were first calculated as in the original study and were used for further analysis.

A metagene score for TEAD-TP63 direct induced and repressed target genes was calculated using GSVA [60] for each sample within each dataset separately. Correlation between TF levels (YAP, TAZ, TP63, and TEAD1-4) and metagene scores were calculated with Pearson correlation.

To assess the association between TEAD-TP63 induced and repressed metagene scores and the histological grades in Beane et al. [5], a linear mixed-effect model was used with the histological grade as the main independent variable (coded as a continuous variable from normal to severe dysplasia/carcinoma in situ), and the patient was adjusted as a random effect using nlme [61]. For Merrick et al., [9] a linear model was used with the histological grade as the main independent variable (coded as a continuous variable from normal to severe dysplasia/carcinoma in situ).

To study the association between TEAD-TP63 induced and repressed target genes and the lesion progression status, a gene rank list was first calculated for each dataset. For Beane et al. [5], genes were ranked by the t-statistic for their association with progression status from a linear mixed effect model, comparing between progressive/persistent lesions and the regressive ones among samples of the Proliferative subtype, adjusting for the patient as a random variable using duplicateCorrelation function from limma [54]. For the Merrick et al. [9], genes were ranked by the t-statistic of a linear model comparing all progressive/persistent samples (including the persistent bronchial dysplasia and progressive non-dysplasia groups in the original annotation) to the regressive ones (regressive bronchial dysplasia group). Then, GSEA [55] was used to test whether the TEAD-TP63 direct induced and repress target genes were enriched within the rank lists.

Immune infiltration scores of 24 immune cell-types within the bulk RNA-seq samples were calculated using GSVA [60] based on the immune cell-type-specific signature genes from Bindea et al. [62, 63]. The association between metagene scores of TEAD-TP63 induced and repressed target and the immune cell-type scores were calculated with Pearson correlation. The association between the immune cell-type scores and lesion progression status was examined using the same model as described above.

### Single-cell RNA-seq data analysis

10X Chromium single-cell RNA-seq datasets of the human healthy airway and normal lung tissue, including normalized count data and annotations, were obtained from Travaglini et al. (EGAS00001004344) [64] and Deprez et al. (EGAS00001004082) [65]. Cell clustering and cell-type annotation from the original studies were used. For Travaglini, the tSNE coordinates across all cells was calculated using the top 20 principal components with 2 K highly-variable genes for visualization using the RunTSNE function from Seurat [66]. For Deprez et al. healthy airway dataset [65], only the proximal and intermediate airway biopsy samples were used for our analysis to match the cellular composition of bronchial premalignant lesion bulk RNA-seq data. UMAP coordinates were obtained from covid19cellatlas.org/index.healthy.html. The metagene scores of TEAD-TP63 direct induced/repressed target gene sets and MHC Class II genes were calculated using AUCell R package [67] based on normalized count data and were compared between cell-types using one-tail Wilcoxon test. Ligand-receptor analysis was performed in Travaglini et al. [64] normal lung dataset using CellChat [68] with the default parameters and nboot = 500.

### Datasets used and code availability

RNA-seq and ChIP Seq datasets have been deposited to NCBI GEO GSE213656 and GSE158307. Human RNA-seq datasets are listed as GSE109743 and GSE114489.

## Results

### TP63 and TEAD expression is elevated in PML histological progression

In prior work, we demonstrated that activation of the transcriptional effectors YAP and TAZ stimulates lung epithelial basal cell growth and induces gene expression associated with progressive bronchial PML [24]. Given the reported association of TP63 with YAP and TAZ and the critical functions of TP63 in maintaining airway basal stem cell identity, we hypothesized that cooperation between YAP/TAZ and TP63 in airway epithelial cells contributes to the progression of premalignant human airway disease and LUSC.

**Fig. 1:**
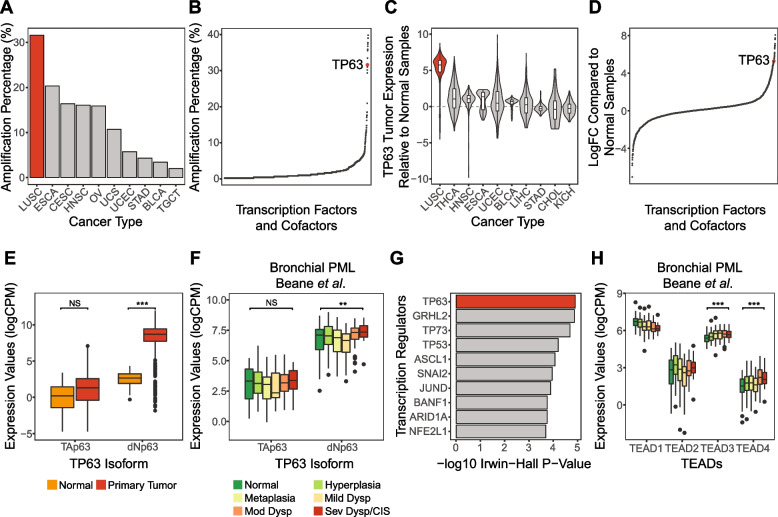
TP63 is associated human LUSC carcinogenesis and early lung cancer progression. **a** TP63 amplification frequency in TCGA samples by cancer types. The top 10 cancer types ranked by TP63 amplification frequencies are shown. **b** Transcription factors ranked by amplification frequencies in the TCGA LUSC samples. The vertical axis showed the amplification frequency for each TF in TCGA LUSC, and the horizontal axis shows the ranking of TFs by the amplification frequency. **c** TP63 expression z-scores in TCGA primary tumor samples relative to normal samples by cancer types. The top 10 cancer types ranked by TP63 overexpression are shown. **d** Transcription factors ranked by logFC comparing TCGA LUSC tumor to normal samples. The vertical axis shows the logFC of each TF and the horizontal axis shows the ranking of TFs by the logFC. **e**–**f** Boxplots show the TP63 isoform (TAp63 and dNp63) expression levels between normal and primary tumor samples in (**e**) TCGA LUSC and (**f**) across bronchial PML histological grades in Beane et al. (Mild/Mod/Sev Dysp = Mild/Moderate/Severe Dysplasia; CIS = carcinoma in situ). *p < 0.05, **p < 0.01, ***p < 0.001. **g** Top 10 TFs responsible for genes upregulated in LUSC versus normal samples based on BART cancer. **h** Boxplots show the TEADs expression levels across bronchial PML histological grades in Beane et al. Only significant increase with higher histological grades are marked. ***p < 0.001

We first sought to examine the association of *TP63* with lung squamous tumor samples. Analysis of LUSC data available from The Cancer Genome Atlas (TCGA) showed that over 30% of tumors have an amplification of *TP63*, which is more frequent compared to other cancers profiled in TCGA (Fig. 1A) and is among the most frequently amplified transcription factors in LUSC (Fig. 1B and Supplementary Table S4). We found that *TP63* is also significantly overexpressed in primary tumor samples compared to normal tissues in TCGA LUSC data compared to other cancer types (Fig. 1C), ranking high among transcription factors expressed in this cancer subtype (Fig. 1D and Supplementary Table S5). Notably, ΔNp63, the major TP63 isoform with oncogenic functions [69], is more highly expressed in primary LUSC tumor samples than the full-length Tap63 (Fig. 1E and Supplementary Fig. 1A; linear model *p*-value < 0.001). Similar high expression of ΔNp63 was observed in high-grade PML samples compared with low-grade PMLs [5] (Fig. 1F and Supplementary Fig. 1B; mixed effect model *p*-value < 0.01), and ΔNp63 was the dominant isoform across all stages of PML samples, suggesting that TP63 activity may be important for high-grade PML development.

To further explore the association between *TP63* and increasing PML histologic grade, we performed TF enrichment among the top 500 genes upregulated in higher grade PML. Results from both Binding Analysis for Regulation of Transcription (BART) [70] and ChIP-X Enrichment Analysis 3 (ChEA3) [71] indicated TP63 as a highly significant TF regulating the genes in this histologic-grade associated gene module (ranked 1st by BART and 18th by ChEA3 MeanRank method; Supplementary Table S6). Notably, TP63 was also listed as the top TF regulating genes that are up-regulated in LUSC compared to normal tissues in BART-Cancer [72] (Fig. 1G; *p*-value < 0.001).

We also found that the expression levels of *TEAD3* and *TEAD4*, which encode transcription factors of the TEAD family that are regulated by YAP/TAZ binding, were significantly increased with higher histologic grades in PML samples [5] (Fig. 1H; mixed effect model *p*-value < 0.001). Similar trends were not observed for TEAD1/2 or YAP/TAZ (Supplementary Fig. 1C). These observations suggested that TP63 and TEAD transcription factors, both of which are linked to YAP/TAZ function, may be associated with bronchial PML progression.

### YAP, TEAD and TP63 bind to the same genomic sites in basal bronchial epithelial cells

To characterize the genes directly regulated by YAP/TAZ, TEAD and TP63, we performed chromatin immunoprecipitation sequencing (ChIP-seq) from proliferating HBECs using antibodies targeting YAP, TEADs (pan-TEAD antibody) and TP63. In total, 4817, 21,925, and 23,692 consensus peaks (overlapped between replicates) were identified for YAP, TEAD, and TP63, respectively. 25% of the peaks from each ChIP-seq experiment were located within 2.5 kb from the gene TSSs or within the promoter regions, while over 50% of the peaks were located more than 10 kb away from the nearest TSSs (Supplementary Fig. 2A-B), indicating potential long-range gene regulation for YAP, TEAD, and TP63, as previously suggested [73, 74].

**Fig. 2:**
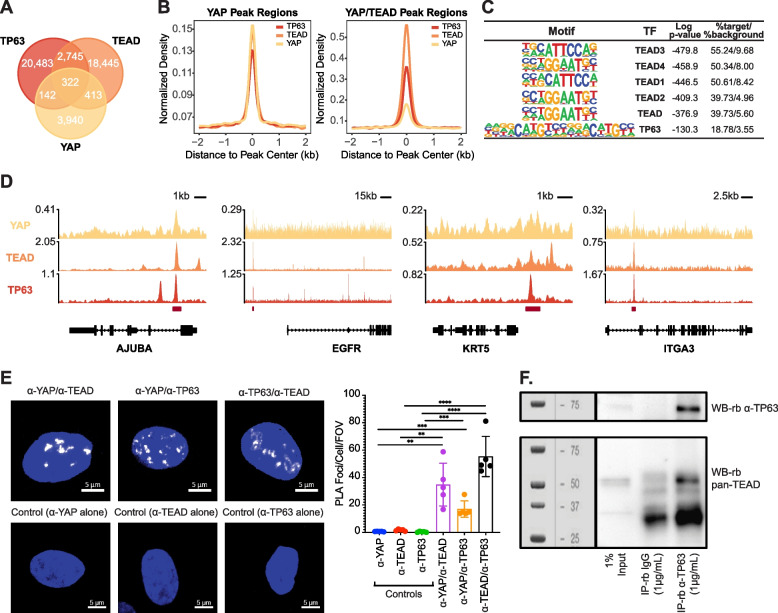
YAP, TP63 and TEADs associate and show common genomic binding regions in HBECs. **a** Venn diagram shows peak overlaps between YAP, TEAD and TP63 chromatin binding domains in HBECs. **b** Distribution of YAP/TEAD/TP63 ChIP-seq signal around ± 2 kb of YAP and YAP/TEAD overlapped peak regions (*N* = 4817 and 735). **c** Top transcription factor binding motifs enriched in the YAP/TEAD overlapped peak regions in HBECs. *P*-values were calculated by HOMER. **d** YAP, TEAD and TP63 ChIP-seq tracks shows the co-binding at the promoter regions of Hippo or TP63 canonical target genes. Overlapped peak regions are highlighted with red strips below. **e** Proximity ligation assays (PLA) demonstrating close association between YAP, TEAD and TP63 in proliferating HBECs. Representative images of association between the respective factors are shown, along with quantification showing the average number of PLA foci per cell across a field of view. The average of five fields of view (FOV) for each condition with standard error of mean is depicted. Unpaired t-test **p < 0.01, *** *p* < 0.001, ****p < 0.0001. **f** Western blot showing TEAD and TP63 co-immunoprecipitation in HBECS

We next compared the chromatin binding patterns of YAP, TEAD, and TP63 and found significant peak overlaps between the three factors: 735 peaks were overlapped between YAP and TEAD, 464 were overlapped between YAP and TP63, and 322 were overlapped between all three (Fig. 2A; permutation test *p*-value < 0.001). Intriguingly, coverage density analysis not only revealed strong TEAD coverage at the YAP binding regions but also TP63 coverage at both the YAP binding and YAP-TEAD co-binding regions (Fig. 2B). HOMER motif enrichment analysis on the YAP-TEAD co-binding peaks identified TEAD and TP63 motifs as the two most significantly enriched TF binding motifs (Fig. 2C; *p*-value < 0.001). Similarly, both TEAD and TP63 motifs were significantly enriched at the YAP binding regions and YAP-TP63 co-binding regions, suggesting these are the primary DNA binding factors mediating YAP function in HBECs (Supplementary Fig. 2C). Regions bound by YAP, TEAD and TP63 included the promoters/enhancers of target genes identified in other contexts, including *AJUBA* for YAP and *EGFR* for TP63, as well as genes associated with basal cell identity, such as *KRT5* and *ITGA3* (Fig. 2D) [75, 76]. These analyses showed highly overlapped chromatin-binding profiles between YAP, TEAD, and TP63, prompting us to test for association between these factors. YAP is documented to interact with TEADs [31, 77] and TP63 [17, 19], which we validated using proximity ligation assays (PLA) [78] in proliferating HBECs (representative PLA in individual cells along with quantitation shown in Fig. 2E and fields of view (FOV) in Supplementary Fig. 2D). We also observed strong association between TEAD and TP63 using PLA, which we further validated using co-immunoprecipitation experiments (Fig. 2F). Taken together these data suggest that YAP, TEAD and p63 form a transcriptional complex in proliferating basal bronchial epithelial cells.

### TP63 and TEAD co-regulate gene expression in the basal bronchial epithelial cells

**Fig. 3:**
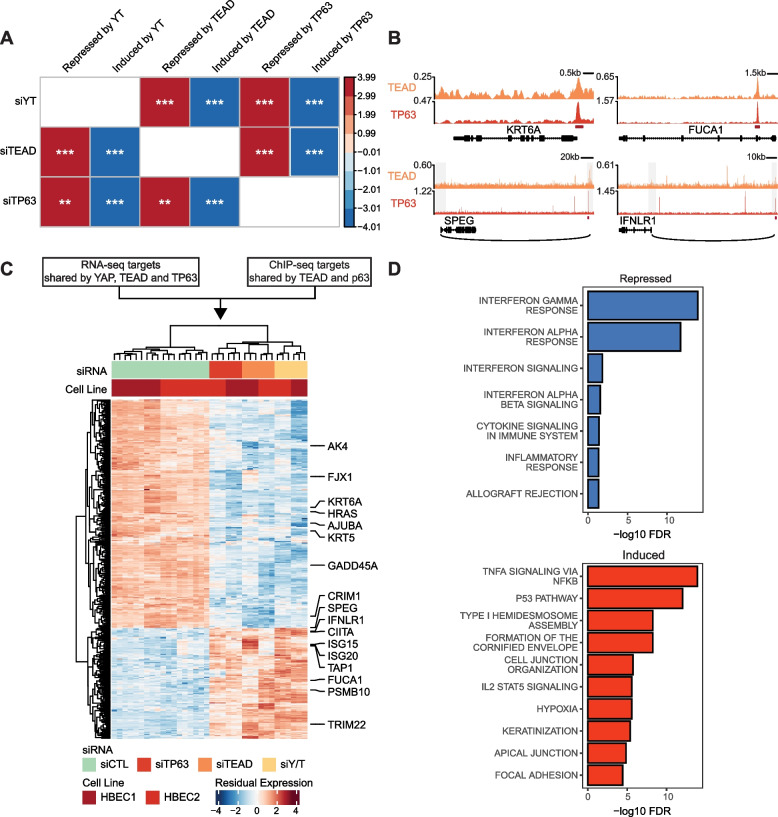
YAP, TP63 and TEADs co-regulate genes associated with carcinogenesis pathways in HBECs. **a** Correlation plot summarizes the GSEA results of genes associated with YAP/TAZ, TEAD and TP63 siRNA treatments in HBECs. Rank lists were generated by arranging genes in order of t-statistics for their association with siRNA treatment comparing to the controls in HBECs. Genes significantly up or down-regulated with the siRNA treatments were used as gene sets (absolute logFC > 0.5 and FDR < 0.05). **p < 0.01, ***p < 0.001. **b** TEAD and TP63 ChIP-seq tracks shows the representative co-binding associated TEAD-TP63 direct target genes. Overlapped peak regions are shown in red strips. The grey rectangles indicate the fragment regions in pcHi-C and the black arcs show the long-range interactions between distal elements and promoter regions. Only the direct target genes are plotted. **c** Heatmap of gene expression significantly altered in siYAP/TAZ, siTEAD and siTP63 treatment (absolute logFC > 0.5 and FDR < 0.05). Genes annotated on the right are associated with interferon response pathways or shown to be canonical target genes of Hippo or TP63 pathways. **d** Top enriched functional pathways associated with the TEAD-TP63 direct repressed (top) and induced (bottom) target genes

To gain insight into the transcriptional relationship between YAP, TEAD, and TP63, we performed bulk RNA sequencing on proliferating HBECs treated with siRNA targeting YAP/TAZ, TEADs, and TP63. Differential expression analysis comparing siRNA treated samples to the controls identified 2581, 2120, and 1566 genes down-regulated in expression following siRNA-mediated knockdown of YAP/TAZ, TEAD, and TP63, respectively (i.e., genes normally induced by these factors). This analysis also identified 2510, 2096, and 1391 genes, that were up-regulated in expression following siRNA-mediated knockdown YAP/TAZ, TEAD, and TP63, respectively (i.e., genes normally repressed by these factors). YAP/TAZ, TEAD, and TP63 induced genes (i.e., genes down-regulated with siRNA-mediated knockdown) were significantly enriched within each other’s respective gene sets, and a similar pattern was observed for YAP/TAZ, TEAD, and TP63 repressed genes (i.e., genes up-regulated with siRNA-mediated knockdown) (Fig. 3A; GSEA *p*-value < 0.01), suggesting that YAP, TEAD and TP63 regulate a shared gene expression program in HBECs.

Next, we sought to identify genes directly co-regulated by YAP, TEAD, and TP63 by integrating the chromatin binding profiles from ChIP-seq experiments with the gene expression profiles from the RNA-sequencing of the siRNA experiments. Since only TEAD and p63 directly bind DNA, and due to higher quality data obtained from our ChIP-seq analysis of TEAD and TP63, we combined our TEAD-p63 overlapped peaks (*N* = 3067) with our RNA-seq analysis to identify potential direct targets. Genes with TSS within 50 kb from the TEAD-TP63 overlapped binding regions or potentially regulated by TEAD and TP63 through long range interactions at distal regions (based on pcHi-C data from Jung et al. [58]) were labeled as direct targets (Fig. 3B). This analysis identified 260 TEAD-TP63 directly induced (i.e., genes with binding peaks that were down-regulated following siRNA-mediated knockdown) and 126 directly repressed (i.e., genes with binding peaks that were up-regulated following siRNA-mediated knockdown) target genes (Fig. 3C and Supplementary Table S7). These represented over 20% of the overlapped differential expressed genes from the three siRNA experiments (Supplementary Fig. 3A). Among the TEAD-TP63 direct induced targets, we found several canonical targets for both the Hippo pathway (AJUBA, GADD45A, FJX1, and CRIM1) [76] and TP63 pathway (AK4, KRT5/6A, and HRAS) [75]. We also observed several interferon response and antigen processing related genes among the TEAD-TP63 direct repressed genes.

To further validate the regulation of target genes via endogenous YAP/TAZ activation, and to test the effects of Hippo pathway regulation of these targets, we depleted the LATS1/2 kinases in HBECs using siRNA and found that TEAD-TP63 directly induced and repressed target genes were among the genes most down- and up-regulated in the siLATS treatment samples compared to the controls (Supplementary Fig. 3B; GSEA *p*-value < 0.005). Functional enrichment analysis revealed that the TEAD-TP63 induced genes are associated with cell proliferation and extracellular matrix-associated pathways, and the repressed genes are strongly enriched for interferon alpha and gamma responses (Fig. 3D; hypergeometric FDR < 0.001).

### The TP63/TEAD repressed gene program is associated with early immune evasion in the bronchial premalignant lesions

**Fig. 4:**
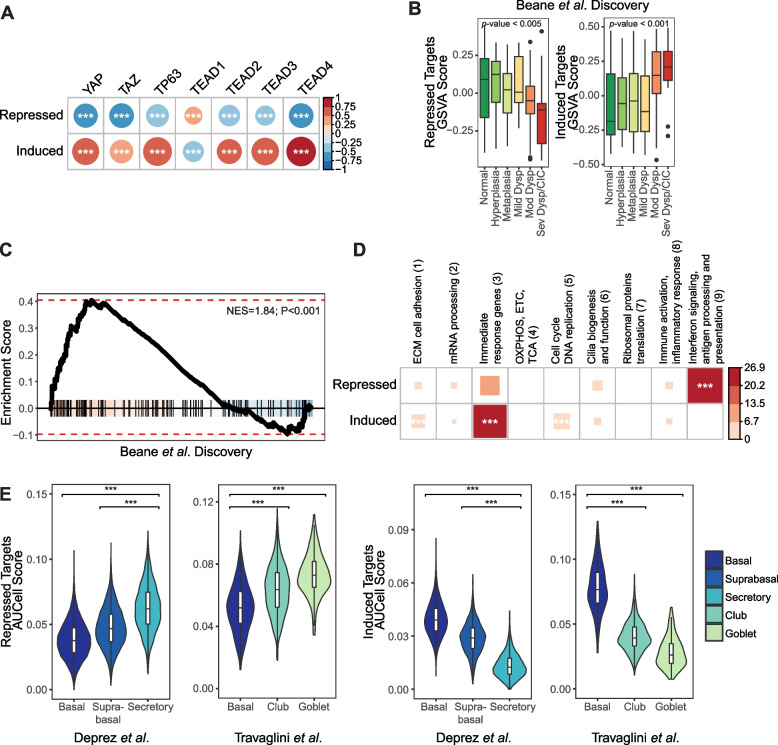
TEAD-TP63 direct target genes are associated with human bronchial PML progressive pathology and early immune evasion. **a** Correlation plot shows the relationship between the expression levels of transcription factors and metagene scores of TEAD-TP63 direct induced and repressed target genes (calculated with GSVA) in the Beane et al. Discovery cohort. The color and the size of the circles indicate the Pearson correlation coefficients. ***Pearson correlation, FDR < 0.005. **b** The metagene scores of TEAD-TP63 direct repressed (left) and induced (right) target gene sets across human bronchial PML data by histological grades in the Beane et al. Discovery cohort (Mild/Mod/Sev Dysp = Mild/Moderate/Severe Dysplasia; CIS = carcinoma in situ). **c** Enrichment plot for TEAD-TP63 direct repressed target genes among genes ranked by tstatistics comparing the regressive PML samples to the progressive/persistent ones of the Proliferative subtypes in the Beane et al. Discovery cohort (positive t-statistics indicate upregulation among the regressive lesions). **d** Bubble plot shows the enrichment of TEAD-TP63 direct repressed and induced target gene sets among human bronchial PML co-expressed gene modules. The color and the size of the squares indicate the odds ratio. ***Fisher’s exact test *p*-value < 0.001. **e** Violin plots show the summarized expression of TEAD-TP63 direct repressed (left) and induced (right) target genes (calculated using AUCell) in the healthy human airway scRNA-seq data from Deprez et al. and human lung scRNA-seq data from Travaglini et al. ***one-tail Wilconxon test, *p*-value < 0.001

To explore the potential relationship between YAP/TAZ, TEAD and p63 transcriptional regulation and the gene expression changes associated with bronchial carcinogenesis, we measured metagene scores of directly induced and repressed target genes shared by these factors in human PML patient endobronchial biopsies. Metagene scores were calculated for the induced and repressed targets separately in three gene expression datasets which examined progressive PML pathology, which included RNA sequencing data from Beane et al., (GSE109743) which defined both a discovery cohort and an independent validation cohort [5] and Affymetrix Gene 1.0 ST microarray data from Merrick et al., (GSE114489) [9]. First, we validated that the expression of TEAD-TP63 direct targets were correlated with the expression levels of *YAP*, *TAZ/WWTR1*, *TEAD*, and *TP63*. A strong positive correlation was observed between the metagene score for the directly induced targets of TEAD-TP63 and *YAP, TAZ/WWTR1, TP63*, and *TEAD2/3/4* (*TEAD1* did not show a similar correlation) in Beane et al. discovery cohort [5], and conversely a negative correlation was observed for the directly repressed targets of TEAD-TP63 (Fig. 4A). Similar correlation patterns were also observed in the Beane et al. validation cohort [5] and the Merrick et al. [9] dataset (Supplementary Fig. 4A).

Notably, TEAD-TP63 direct target genes were significantly associated with increased PML histologic severity; the metagene score of the induced targets were significantly increased in higher grade PML samples (linear model *p*-value < 0.001 in all three datasets) and the metagene score of the repressed targets were decreased, although less significantly (Fig. 4B and Supplementary Fig. 4B). TEAD-TP63 directly repressed target genes were significantly enriched among the genes down-regulated in progressive/persistent compared with regressive PMLs among the samples of Proliferative subtype described in the Beane et al. discovery (GSEA *p*-value < 0.001) and validation cohort (GSEA *p*-value < 0.05) [5], and among all samples in Merrick et al. (GSEA *p*-value < 0.001) [9] (Fig. 4C and Supplementary Fig. 4C). Directly induced genes were also strongly enriched among the genes up-regulated in progressive/persistent PMLs in the Merrick et al. cohort [9] (Supplementary Fig. 4C; GSEA *p*-value < 0.001), although this enrichment was not as clear in the Beane et al. data [5]. Collectively, these observations suggest that shared TEAD and p63 activities are associated with precancerous airway disease progression.

To gain functional insight into TEAD-TP63-regulated genes, we explored potential associations with gene modules identified from network analyses of PML data from prior work, which revealed significant overlap between TEAD-TP63 direct induced target genes and three co-expressed gene modules (Modules 1, 3, and 5) described in Beane et al. [5]. TEAD-TP63 targets in these modules were enriched for genes associated with extracellular matrix/cell adhesion, immediate response, and cell-cycle/DNA-replication pathways, respectively (Fig. 4D; Fisher’s exact test *p*-value < 0.001), suggesting these gene networks are induced by TEAD-P63 in bronchial PMLs. We also observed a significant overlap between the TEAD-TP63 direct repressed target genes and co-expressed gene module (Module 9) from Beane et al. [5], which is enriched for genes encoding antigen presentation and interferon response pathways factors, strongly associated with PML progressive pathology and is correlated with the level of immune cell infiltration, including cytotoxic cells, CD8 + T cells, NK cells, Th1 CD4 + T cells, and activated dendritic cells.

Previous studies have suggested immune regulatory functions reside in distinct subsets of airway epithelial cells, with airway secretory cells playing key roles in promoting lymphocytic infiltration [79, 80]. We therefore examined the cell-type expression of the genes directly induced or repressed by TEAD-TP63 by calculating the metagene score of TEAD-TP63 direct induced and repressed target genes with AUCell [67] in two normal human airway/lung single-cell RNA-seq datasets, from Deprez et al., [65] (*N* = 41,134) and Travaglini et al., [64] (*N* = 65,662). High expression of genes directly induced by TEAD-TP63 was observed within the basal and suprabasal epithelial cell subsets, while repressed target genes were expressed at lower levels in these same subsets (Fig. 4E and Supplementary Fig. 4D Wilcoxon one-tail test *p*-value < 0.001). These observations suggest cooperation between YAP/TAZ, TEAD, and TP63 in basal and suprabasal cells.

### YAP/TAZ-TEAD-TP63 down-regulate major histocompatibility complex factors transactivator CIITA in bronchial epithelial cells

**Fig. 5:**
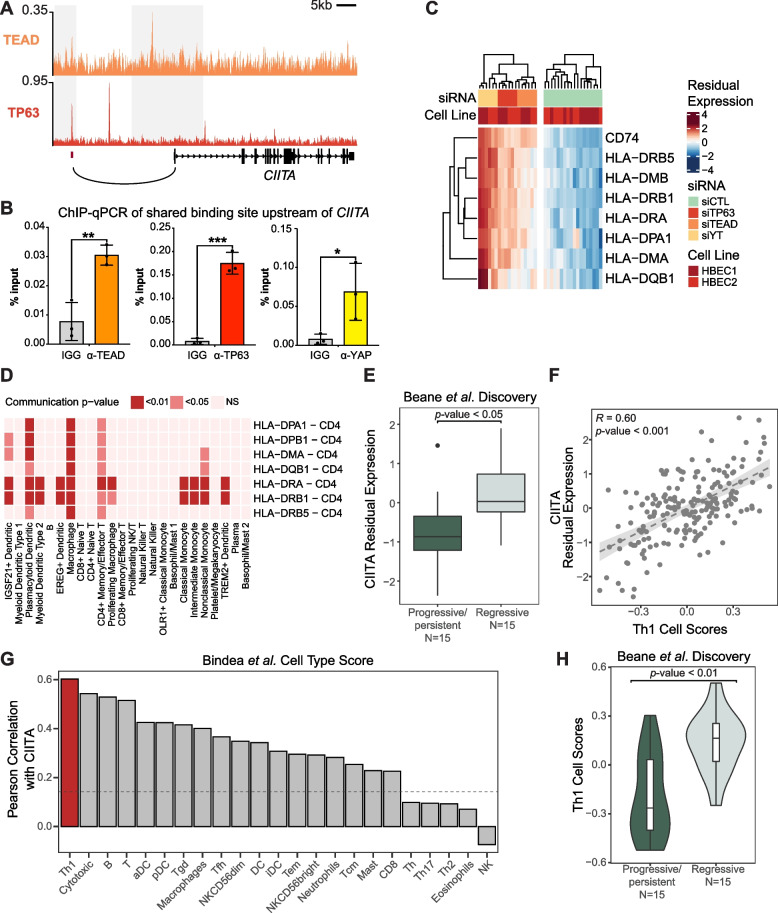
CIITA associates with bronchial PML progressive pathology and tracks with MHC Class II gene expression and the presence of Th1 T cells. **a** TEAD and TP63 ChIP-seq tracks showing the co-binding peaks associated with *CIITA*. Overlapped peak regions are shown with red strips below. The grey rectangles indicate the fragment regions in pcHi-C and the black arc depicts the interactions between distal elements and CIITA promoter. **b** Proliferating HBECs were lysed and ChIP-qPCR was performed using TEAD, TP63, YAP or IGG control antibodies examining the peak region identified by ChIP-seq as overlapping for TEAD and TP63 binding upstream of *CIITA*. The average % input from three experiments is shown -/ + standard error of mean. Unpaired t-test, *p < 0.01, **p < 0.001, ***p < 0.0001. **c**. Heatmap of significantly repressed MHC Class II gene expression (FDR < 0.01) in siYAP/TAZ, siTEAD and siTP63 treatment in HBECs. **d** Heatmap of communication significance levels between MHC Class II genes in basal cell and binding partners in immune cells in the human lung scRNA-seq data from Travaglini et al*.* The ligand-receptor pairs that involve MHC Class II gene expression in the basal cells are plotted as the row, and the immune cell types that the basal cells are communicating to are plotted as the columns. The color of heatmap reflects the significance levels of the cell–cell communication based on CellChat. **e** Expression levels of CIITA in progressive/persistent and regressive PML samples of the Proliferative subtype in the Beane et al. Discovery cohort. **f** Scatter plots show the Pearson correlation between the expression level of CIITA and Th1 scores (calculated using GSVA based on genes from Bindea et al.) in the Beane et al. Discovery cohort. **g** Immune cell-type ranked by their Pearson correlation coefficients with CIITA expression level in the Beane et al. Discovery cohort. The dashed line indicates the Pearson correlation coefficient that reaches *p*-value = 0.05. **h** Th1 cell scores in progressive/persistent and regressive PML samples of the Proliferative subtype in the Beane et al. Discovery cohort

To explore potential basal cell-immune crosstalk downstream of TEAD-TP63 activity we further examined the target genes, and found CIITA, a MHC Class II transactivator that plays critical functions in inducing the expression of MHC-II related genes [81, 82], as a TEAD-TP63 direct repressed target gene. An overlapping binding site was observed for TEAD and TP63 in a region upstream of the CITTA start site that was identified as a potential distal regulatory region by pcHi-C [58] (Fig. 5A). Using ChIP-qPCR experiments we validated that TEAD and TP63 were enriched at this upstream regulatory element, and further found that YAP was also enriched at this same genomic location in HBECs (Fig. 5B). Given the functions of CIITA, we hypothesized that YAP/TEAD/TP63-mediated repression of CIITA would result in reduced MHC Class II gene expression that translates to immune evasion. Accordingly, we found that most of the genes encoding MHC II family factors were induced following YAP/TAZ, TEAD, or p63 knockdown, indicating repression of MHC family gene expression by YAP/TAZ, TEAD and TP63 (Fig. 5C; linear model FDR < 0.05), including various HLA class II histocompatibility antigens, and CD74, the HLA-DR antigens-associated invariant chain that plays essential roles in the formation and transport of the MHC class II complex [83]. To test a potential relationship with immune evasion, we used CellChat [68] to investigate MHC Class II related ligand-receptor signaling within lung single-cell RNA-seq data from Travaglini et al. [64], which is a dataset with detailed annotation of immune cell subsets. 61,344 significant ligand-receptor interactions between 57 cell-types were identified (*p*-value < 0.05), and 1214 ligand-receptor interaction pairs involving ligands expressed on basal cells. Among these were 41 interactions between MHC class II genes expressed in basal cells and CD4 in various immune cells (Fig. 5D), including mature CD4 + T cells, dendritic cells and macrophages.

CIITA belongs to the antigen presentation/interferon response co-expressed gene module (Module 9) previously identified in Beane et al. [5] as being down-regulated amongst progressive/persistent Proliferative subtype PMLs (Fig. 5E; linear model *p*-value < 0.05). Similar association between lower CIITA expression and PML progression was observed in the Beane et al. validation (Supplementary Fig. 5A; p-value 0.45) and in Merrick et al. datasets [9] (*p*-value < 0.05). Concordantly, most of the MHC Class II genes were strongly down-regulated among the progressive/persistent PMLs across three datasets (Supplementary Table S8). These data therefore suggest that repression of CIITA mediated MHC Class II expression by YAP/TAZ-TEAD-TP63 is associated with early immune-evasion and PML progression.

In previous work by Merrick et al. [9], increased epithelial MHC Class II molecule HLA-DRA expression had been associated with a regressive PML phenotype and associated with elevated expression of Th1 marker genes. Similarly, ligand-receptor analysis showed potential communication between bronchial basal population and CD4 + T cells utilizing MHC Class II and CD4 interactions. Hence, we sought to further quantify the association between CIITA expression and markers of Th1 cells in PML human patient data. Our analysis showed a strong correlation between the expression level of CIITA and Th1 cell-type score, calculated using signature genes from Bindea et al. [62] (Fig. 5F and Supplementary Fig. 5B; Pearson correlation *p*-value < 0.001) with the correlation between CIITA and Th1 being strongest compared to other immune cell-types in data from Beane et al. discovery cohort [5] (Fig. 5G and Supplementary Fig. 5C). Consistently, we observed that the Th1 score is decreased in the progressive/persistent PMLs among the samples of the Proliferative subtype PMLs in Beane et al. [5] discovery (Fig. 5H and Supplementary Fig. 5D; linear model, *p*-value < 0.01) and validation cohorts (*p*-value = 0.13), and among all samples in Merrick et al.^9^ (*p*-value = 0.30), suggesting that decreased Th1 infiltration is predictive of PML progression. We observed that MHC Class II genes are strongly expressed among the secretory epithelial cells in two lung scRNA-seq datasets (Supplementary Fig. 5E), highlighting the role of secretory cells in antigen presentation and suggesting that epithelial expression of MHC II genes is associated with lower Th1 infiltration. Taken together, our observations suggest that YAP/TAZ-TEAD-TP63 activity in bronchial epithelial cells repress MHC Class II genes, potentially through down-regulating CIITA, which contributes to PML progression in part by suppressing the local presence of Th1 cells.

## Discussion

**Fig. 6:**
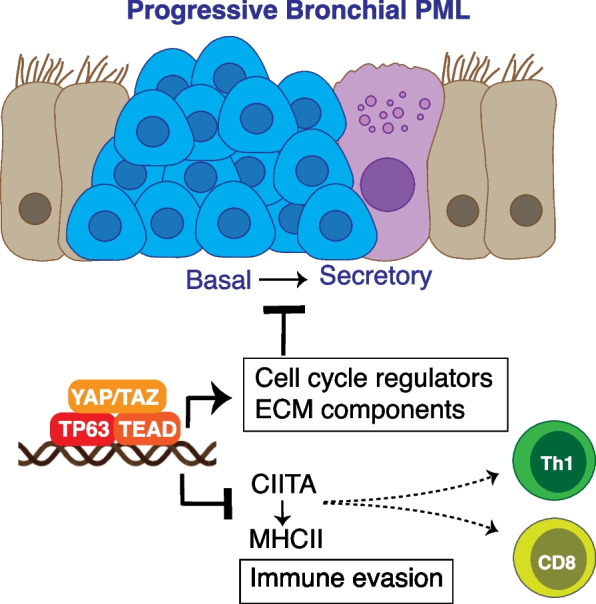
Model for YAP/TAZ-TEAD-TP63 transcriptional complexes in bronchial PMLs. YAP/TAZ, TEAD and TP63 associate on genomic regions that control gene expression important for the growth of bronchial basal cells and the evasion of adaptive immune responses. CITIIA, a key regulator of MHCII genes, is repressed by YAP/TAZ-TEAD-TP63, and CITIIA repression is associated with decreased CD8 T cell recruitment and decreased Th1 T cell responses that track with PML progression

Our study demonstrates that the activity of a gene expression program that is cooperatively regulated by the TEAD and TP63 transcription factors is increased in progressive bronchial PMLs, and that these factors are modulated by the transcriptional effectors YAP and TAZ (biological hypothesis depicted in Fig. 6). Our observations suggest that these factors assemble as a transcriptional complex in bronchial basal cells, as YAP, TEAD and TP63 interact, occupy similar chromatin binding sites and control a conserved gene expression program that strongly associates with PML progression. We mapped genes directly regulated by TEAD and TP63 by ChIP-seq and examined the gene expression consequences of siRNA-mediated gene silencing using RNA-seq. Directly induced genes of TP63 and TEAD encode factors involved in cell proliferation and extracellular matrix production, while directly repressed genes include genes associated with interferon downstream signaling and antigen presentation pathways. Our analysis of directly repressed TEAD-TP63 targets showed a particularly strong association between these immune modulating target genes and genes downregulated in the progressive PMLs, suggesting that TEAD-TP63 activity modulates immune function in the lung. Notable genes directly regulated by TEAD-TP63 included *CIITA,* which encodes a transcriptional transactivator that functions as a key regulator of MHC Class II genes. Our analyses across several datasets demonstrated that low *CIITA* expression is associated with progressive PML pathology and is negatively correlated with genes associated with Th1 cell activity, suggesting that epithelial control of MHC presentation by YAP-TEAD-TP63 modifies immune cell responses in early cancer development.

YAP and TP63 have been reported to associate in several contexts, including airway epithelial cells [17–19], and the oncogenic functions of YAP/TAZ rely on their association with the TEAD family of transcription factors [30, 31]. The physical and functional association of TEAD and TP63, suggest that the activity of YAP/TAZ-TEAD in basal cells is mediated by TP63, potentially directing the context of this complex to control lineage specific events. Our observations suggest that the cooperative activity of YAP/TAZ, TEAD, and TP63 induce the expression of genes that promote basal cell proliferation, as well as extracellular matrix components that may be supportive of basal cell self-renewal. Consistent with a pro-proliferative role, genes co-regulated by YAP/TAZ, TEAD and TP63 were strongly enriched among the genes in a proliferation-related co-expression module that was previously shown to be elevated in higher grade PML samples [5]. The regulation of such genes is consistent with observations that induced nuclear YAP/TAZ activity in mouse basal cells promotes basal cell expansion in vivo [24]; and observations that deletion of TP63 results in a loss of basal cells from the airways of mice [11]. Further, TP63 is frequently amplified in squamous cell carcinoma, and increased TP63 has been linked to YAP activation and alteration of TEAD binding [21, 84]. Therefore, these data along with our observations suggest that increased association between YAP, TEAD and TP63 control key processes for the early expansion and eventual progression of bronchial PMLs. However, it is worth noting that some prior observations have suggested that the ectopic over-expression of YAP can repress the growth of LUSC cells growing in vitro and inhibit the expression of TP63 [42], which conceptually opposes the convergence of YAP-TP63 activity in the tumorigenic program. Unlike our study, these conclusions were made using polyploid cancer cells that possess spectrum of mutations, which potentially may uncouple events that occur at the early stage of LUSC development. Alternatively, it is possible that over-expressed YAP may not properly receive signals required for association with TP36 and/or drive DNA-binding events not normally regulated at an endogenous level. Thus, given that our study was conducted in primary HBECs and with primary human PML data we put forward a model in which YAP, TEAD and TP63 cooperatively contribute to the etiology of LUSC.

One key function that we propose is regulated by YAP/TAZ-TEAD-TP63 synergy is the repression of immune modulating factors that may in turn lead to immune evasion. Previous studies have suggested that decreased levels of immune surveillance, particularly decreased interferon responses and antigen processing/presentation, is associated with progressive/persistent PMLs [5, 8, 9]. We found YAP/TAZ, TEAD, and TP63 repress many of the genes linked to immune surveillance in bronchial PMLs, including direct repression of genes involved in interferon response and antigen presentation pathways. Mechanisms for YAP/TAZ-TEAD-mediated transcriptional repression have been described, including the recruitment of the NuRD [85–87], NCoR [88], and Polycomb [89, 90] repressor complexes to regulated genes. Such active repression may be occurring in cells expanding in bronchial PMLs context, and thus additional study of these mechanisms may offer opportunities to reactivate important signals for PML treatment.

The identification of the MHC II transactivator CIITA as a YAP/TAZ-TEAD-TP63 target gene was notable, particularly given the reported low expression of MHC class II genes in progressive bronchial PML [8, 9] and the similar decreases observed in CIITA and MHC class II gene expression with poor immunotherapy responses in melanoma patients and in a rat model of breast cancer [91, 92] and with promoting intestinal tumorigenesis [93]. Moreover, higher MHC Class II gene expression has been suggested as prognosis marker for colorectal carcinoma and triple-negative breast cancer survival [94, 95]. Thus, the ability for YAP/TAZ-TEAD-TP63 to repress the expression of CIITA and MHC class II molecules in expanding basal epithelial cells may be a key mechanism for how early PMLs evade immune clearance.

Interestingly, many of the genes repressed by YAP/TAZ, TEAD, and TP63, including the MHC Class II genes, were highly expressed in airway secretory cells. This raises interesting questions about how the composition of the bronchial epithelium might influence immune-surveillance. Lung club cells have been shown to be crucial for the efficacy of radiation and immune checkpoint inhibitor combined therapy for non-small cell lung cancer [80], and MHC class II expressing lung epithelial cells act as antigen-presenting cells to direct CD4 + T helper cell functions [79]. Thus, increased YAP/TAZ-TEAD-TP63 activity that favors the basal cell state would be associated with less immune infiltration and a worse prognosis in PMLs. Interestingly, similar stem-cell-like populations with high developmental plasticity and proliferation potential have been observed in adenocarcinoma and metastatic lung cancers [96, 97], suggesting possible similar mechanisms that couple cell fate with immune control.

## Conclusions

Our study maps the transcriptional landscape that is regulated by the Hippo pathway effectors YAP/TAZ and the TEAD and P63 transcription factors in human bronchial epithelial cells. Our results identify functional convergence of these factors on regulatory elements of genes that associate with early pre-malignant stages of lung cancer, which notably includes the repression of genes encoding immune-regulatory factors. Thus, the synergistic transcriptional activity of YAP/TAZ, TEADs, and P63 likely contributes to the immune evasive microenvironment associated with progressive PMLs and offers a means to means to identify bronchial PMLs with the potential to progress. Finally, targeting the YAP/TAZ-TEAD-TP63 complex may provide a therapeutic opportunity for intercepting early lung carcinogenesis, which is something that may be feasible given recent efforts that have been devoted towards developing YAP/TAZ-TEAD inhibitors [26].

## Supplementary Information

**Additional file 1:** **Supplementary Table S1.** HBEC patient info. **Supplementary Table S2.** siRNA information. **Supplementary Table S3.** ChIP-qPCR primers. **Supplementary Table S4.** TF amplification frequencies in TCGA LUSC. **Supplementary Table S5.** TF overexpression in TCGA LUSC compared to normal samples. **Supplementary Table S6.** Top enriched TFs by BART and ChEA3 among the top 500 genes upreguatled with histological grades (BART results were ranked by Irwin Hall *p*-value. ChEA3 results were ranked by the MeanRank results). **Supplementary Table S7.** Differential expression results for the TEAD-TP63 directly regulated genes. **Supplementary Table S8.** Differential expression results for the MHC II genes (shown in Figure 5C) by progression status in PML datasets.

**Additional file 2:** **Supplementary Figure 1.** TP63 isoform expression levels in TCGA-LUSC and in bronchial PML biopsy data related to Figure 1. **Supplementary Figure 2.** ChIP-seq analysis of YAP/TEAD/TP63 chromatin binding profiles related to Figure 2. **Supplementary Figure 3.** Transcriptomic analysis of TEAD-TP63 direct regulated target genes related to Figure 3. **Supplementary Figure 4.** Transcriptomic analysis of TEAD-TP63 direct regulated target genes in human bronchial PML data and lung scRNA-seq data related to Figure 4. **Supplementary Figure 5.** Analysis of CIITA in human bronchial PML data and lung scRNAseq data related to Figure 5.

## Data Availability

The datasets used and/or analyzed in the study are publicly available and all materials used are available upon request.

## Abbreviations

ChIP: Chromatin Immunoprecipitation
CIITA: Class II Major Histocompatibility Complex Transactivator
FDR: False Discovery Rate
GSEA: Gene Set Enrichment Analysis
HBECs: Human bronchial epithelial cells
LUSC: Lung squamous cell carcinoma
MHC: Major histocompatibility complex
PCR: Polymerase chain reaction
PLA: Proximity ligation assay
PMLs: Premalignant lesions
siRNA: Small interfering RNA
TAZ: Transcriptional co-activator with PDZ-binding motif
TCGA: The Cancer Genome Atlas
TEAD: TEA domain (transcription factor)
YAP: Yes-associated protein
