# Supplementary material for: Convergence of YAP/TAZ, TEAD and TP63 activity is associated with bronchial premalignant severity and progression
Source: J Exp Clin Cancer Res. 2023 May 8;42:116. doi: 10.1186/s13046-023-02674-5 (PMC10165825; doi:10.1186/s13046-023-02674-5)
Supplement: Supplementary file 2 — Additional file 2: Supplementary Figure 1. TP63 isoform expression levels in TCGA-LUSC and in bronchial PML biopsy data related to Figure 1. Supplementary Figure 2. ChIP-seq analysis of YAP/TEAD/TP63 chromatin binding profiles related to Figure 2. Supplementary Figure 3. Transcriptomic analysis of TEAD-TP63 direct regulated target genes related to Figure 3. Supplementary Figure 4. Transcriptomic analysis of TEAD-TP63 direct regulated target genes in human bronchial PML data and lung scRNA-seq data related to Figure 4. Supplementary Figure 5. Analysis of CIITA in human bronchial PML data and lung scRNAseq data related to Figure 5. [file 13046_2023_2674_MOESM2_ESM.zip › Supp1.pdf]

Supplementary Figure S1

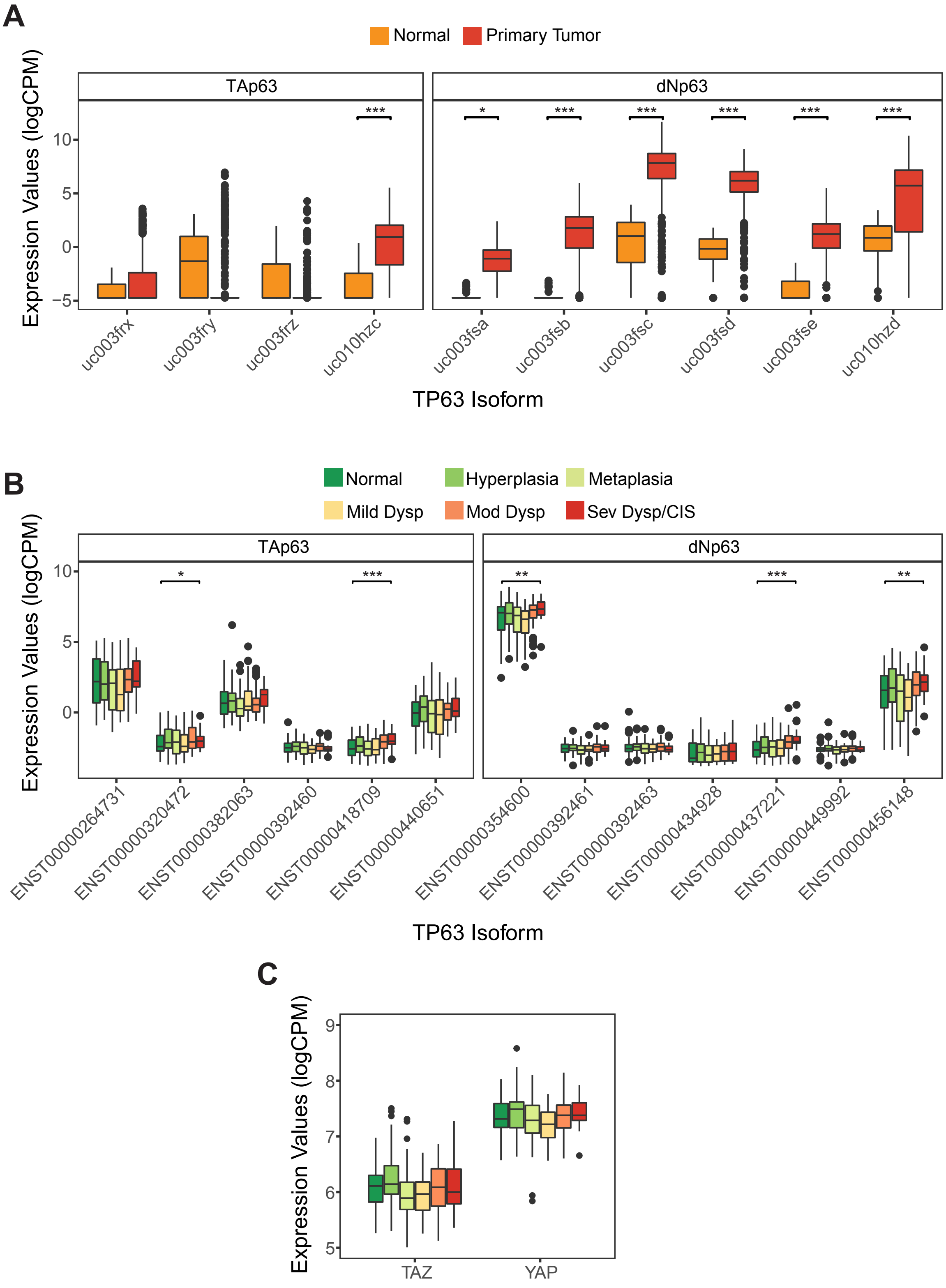

Supplementary Figure 1. TP63 isoform expression levels in TCGA-LUSC and in bronchial PML biopsy data related to Figure 1.

a. Boxplots show all TP63 transcript expression levels between normal and primary tumor samples in TCGA LUSC. Only those with significant increase in primary tumors are marked. $^*p < 0.05$, $^{**}p < 0.01$, $^{***}p < 0.001$.

b. Boxplots show all TP63 protein-coding transcript expression levels across bronchial PML histological grades in Beane *et al*. (Dysp = Dysplasia; CIS = carcinoma *in situ*). $^*p < 0.05$, $^{**}p < 0.01$, $^{***}p < 0.001$.

c. Boxplots show YAP and TAZ expression levels across bronchial PML histological grades in Beane *et al*.
