# Supplementary material for: Convergence of YAP/TAZ, TEAD and TP63 activity is associated with bronchial premalignant severity and progression
Source: J Exp Clin Cancer Res. 2023 May 8;42:116. doi: 10.1186/s13046-023-02674-5 (PMC10165825; doi:10.1186/s13046-023-02674-5)
Supplement: Supplementary file 2 — Additional file 2: Supplementary Figure 1. TP63 isoform expression levels in TCGA-LUSC and in bronchial PML biopsy data related to Figure 1. Supplementary Figure 2. ChIP-seq analysis of YAP/TEAD/TP63 chromatin binding profiles related to Figure 2. Supplementary Figure 3. Transcriptomic analysis of TEAD-TP63 direct regulated target genes related to Figure 3. Supplementary Figure 4. Transcriptomic analysis of TEAD-TP63 direct regulated target genes in human bronchial PML data and lung scRNA-seq data related to Figure 4. Supplementary Figure 5. Analysis of CIITA in human bronchial PML data and lung scRNAseq data related to Figure 5. [file 13046_2023_2674_MOESM2_ESM.zip › Supp2.pdf]

Supplementary Figure S2

A

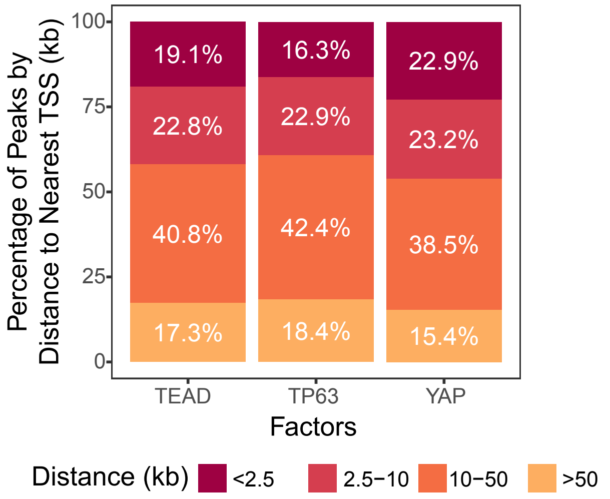

B

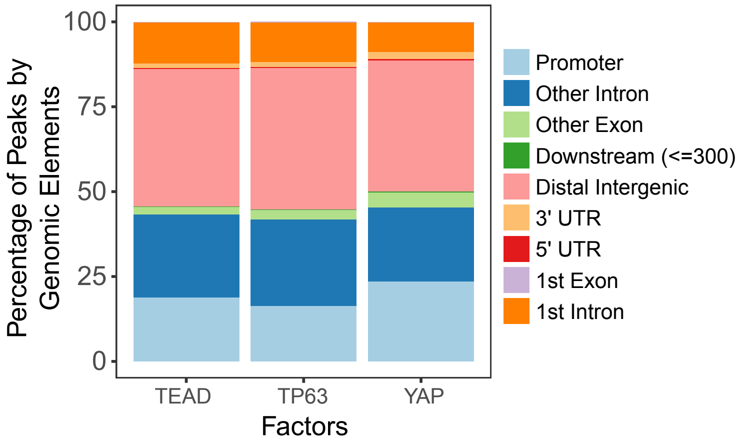

C

Motif	TF	Log p-value	%target/ %background
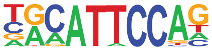	TEAD3	-171.1	17.77/9.27
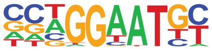	TEAD4	-158.0	15.24/7.67
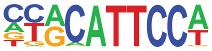	TEAD1	-144.8	15.61/8.22
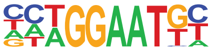	TEAD	-141.0	11.54/5.38
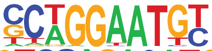	TEAD2	-132.1	10.44/4.78
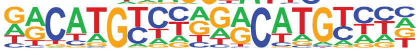	TP53	-74.1	2.64/0.74
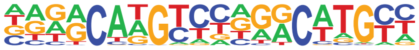	TP73	-63.7	1.76/0.40
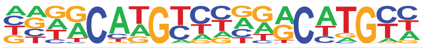	TP63	-59.7	6.60/3.46

Motif	TF	Log p-value	%target/ %background
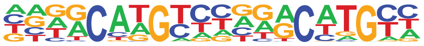	TP63	-366.9	45.47/4.13
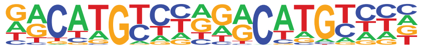	TP53	-224.5	21.55/1.01
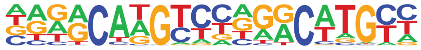	TP73	-209.8	16.59/0.47
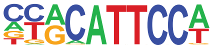	TEAD1	-150.7	36.64/8.08
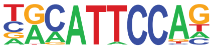	TEAD3	-146.1	38.58/9.33
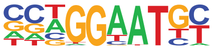	TEAD4	-145.0	35.34/7.77
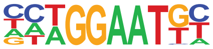	TEAD	-114.7	26.51/5.29
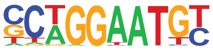	TEAD2	-113.8	25.22/4.78

D

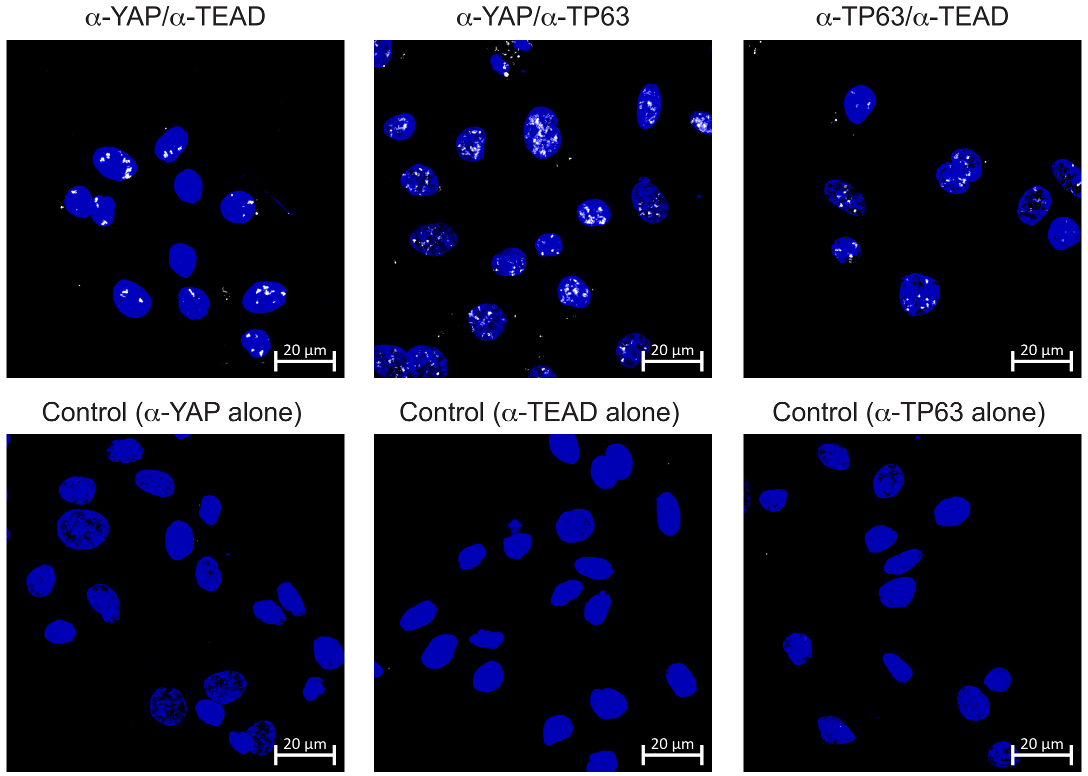

Supplementary Figure 2. ChIP-seq analysis of YAP/TEAD/TP63 chromatin binding profiles related to Figure 2.

a. Distribution of YAP/TEAD/TP63 peaks by distance between peak locations and nearest TSS.

b. Distribution of YAP, TEAD or TP63 peaks by the genomic elements.

c. Top transcription factor binding motifs enriched in the YAP (left) and YAP/TP63 (right) co-binding sites in HBECs. Only unique motifs are shown. P-values were calculated by HOMER.

d. Representative fields of view for respective PLA images that were quantified in Figure 2E. The single antibody controls are shown in the bottom row of images and the interaction conditions are shown in the top row.
