# Supplementary material for: Convergence of YAP/TAZ, TEAD and TP63 activity is associated with bronchial premalignant severity and progression
Source: J Exp Clin Cancer Res. 2023 May 8;42:116. doi: 10.1186/s13046-023-02674-5 (PMC10165825; doi:10.1186/s13046-023-02674-5)
Supplement: Supplementary file 2 — Additional file 2: Supplementary Figure 1. TP63 isoform expression levels in TCGA-LUSC and in bronchial PML biopsy data related to Figure 1. Supplementary Figure 2. ChIP-seq analysis of YAP/TEAD/TP63 chromatin binding profiles related to Figure 2. Supplementary Figure 3. Transcriptomic analysis of TEAD-TP63 direct regulated target genes related to Figure 3. Supplementary Figure 4. Transcriptomic analysis of TEAD-TP63 direct regulated target genes in human bronchial PML data and lung scRNA-seq data related to Figure 4. Supplementary Figure 5. Analysis of CIITA in human bronchial PML data and lung scRNAseq data related to Figure 5. [file 13046_2023_2674_MOESM2_ESM.zip › Suppl3.pdf]

Supplementary Figure S3

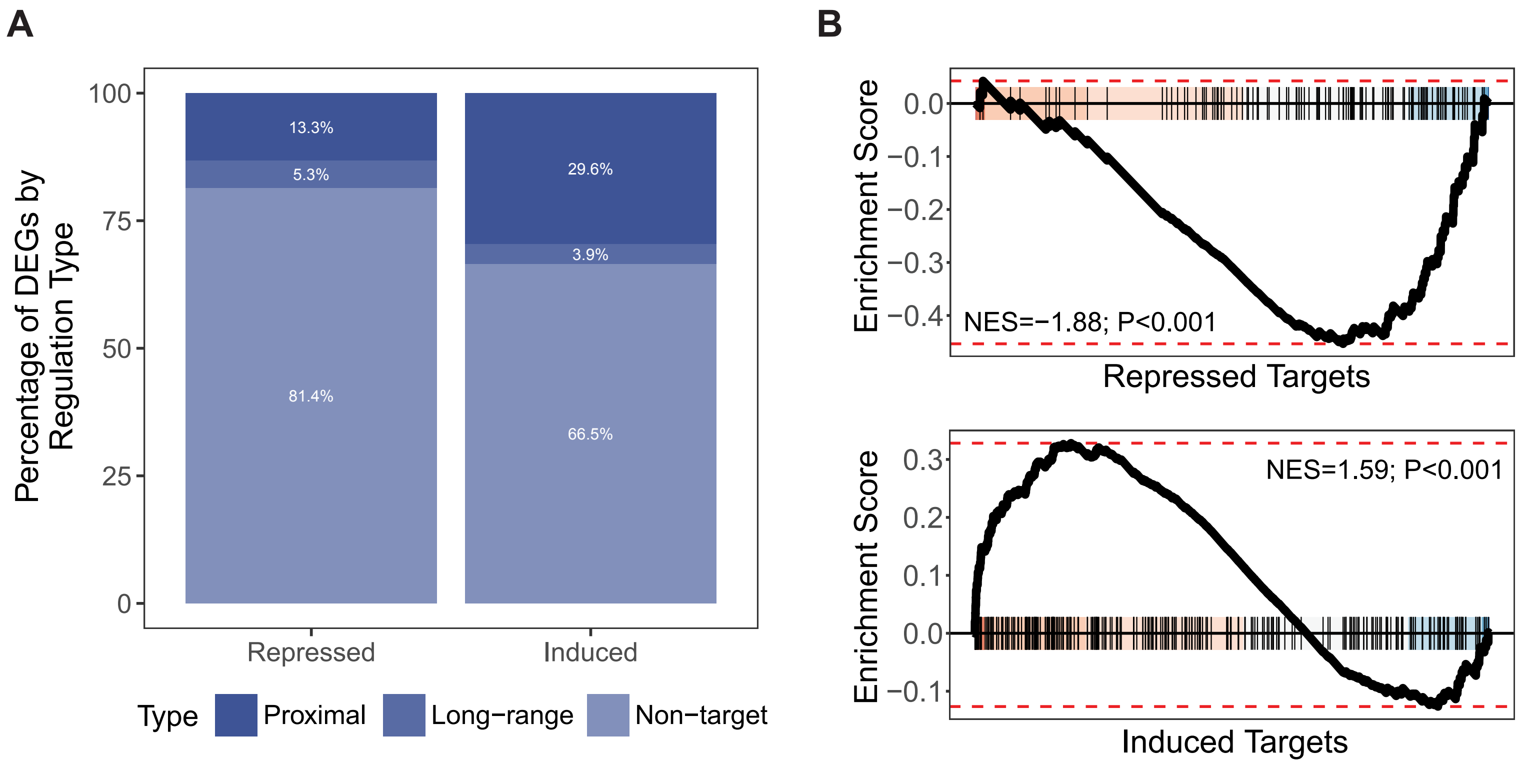

Supplementary Figure 3. Transcriptomic analysis of TEAD-TP63 direct regulated target genes related to Figure 3.

a. Percentage of direct target genes among the overlapped differential expressed genes across siYT, siTEAD and siTP63 experiments. Proximal targets were the genes whose TSS was within 50kb of the TEAD-TP63 overlapped peak regions, long-range targets were associated to overlapped peak through long-range chromosome interaction, and non-target genes were the differential expressed genes that did not meet above criteria.

b. Enrichment plots for TEAD-TP63 repressed (top) and induced (bottom) target genes among genes ranked by t-statistic for their association with siLATS treatment in HBECs (GSEA; p-value <0.005).
