# Supplementary material for: Convergence of YAP/TAZ, TEAD and TP63 activity is associated with bronchial premalignant severity and progression
Source: J Exp Clin Cancer Res. 2023 May 8;42:116. doi: 10.1186/s13046-023-02674-5 (PMC10165825; doi:10.1186/s13046-023-02674-5)
Supplement: Supplementary file 2 — Additional file 2: Supplementary Figure 1. TP63 isoform expression levels in TCGA-LUSC and in bronchial PML biopsy data related to Figure 1. Supplementary Figure 2. ChIP-seq analysis of YAP/TEAD/TP63 chromatin binding profiles related to Figure 2. Supplementary Figure 3. Transcriptomic analysis of TEAD-TP63 direct regulated target genes related to Figure 3. Supplementary Figure 4. Transcriptomic analysis of TEAD-TP63 direct regulated target genes in human bronchial PML data and lung scRNA-seq data related to Figure 4. Supplementary Figure 5. Analysis of CIITA in human bronchial PML data and lung scRNAseq data related to Figure 5. [file 13046_2023_2674_MOESM2_ESM.zip › Suppl4.pdf]

Supplementary Figure S4

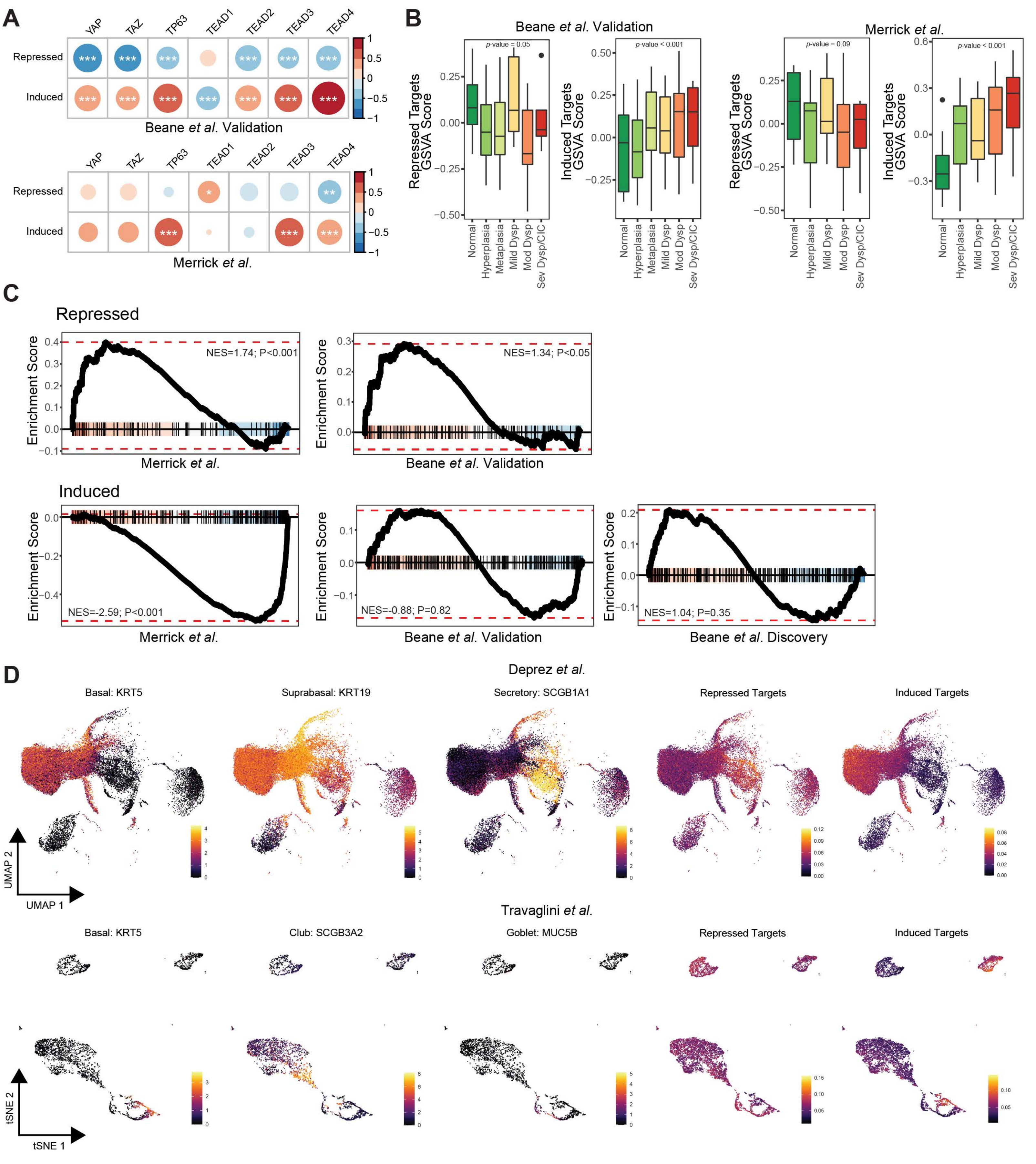

Supplementary Figure 4. Transcriptomic analysis of TEAD-TP63 direct regulated target genes in human bronchial PML data and lung scRNA-seq data related to Figure 4.

a. Correlation plot shows the correlation between the expression levels of transcription factors and metagene scores of TEAD-TP63 direct induced and repressed target genes (calculated with GSVA) in Beane *et al*. Validation cohort (top) and Merrick *et al*. (bottom). The color and the size of the circles indicate the Pearson correlation coefficients. *FDR < 0.05, **FDR < 0.01, ***FDR < 0.005.

b. The metagene scores of TEAD-TP63 direct repressed and induced target gene sets across human bronchial PML data by histological grades in Beane *et al*. Validation cohort (left) and Merrick *et al*. (right).

c. Enrichment plot for TEAD-TP63 direct repressed (top) and induced (bottom) target genes among genes ranked by t-statistics comparing the regressive PML samples to the progressive/persistent ones of the Proliferative subtypes in the Beane *et al*. Discovery/Validation cohort or comparing regressive to progressive/persistent bronchial PML samples in Merrick *et al* (positive t-statistics indicate upregulation in regressive lesions).

d. (Top) UMAP plots show the cell-type marker genes and AUCell scores for TEAD-TP63 direct induced/repressed target gene sets in the healthy human airway scRNA-seq data from Deprez *et al*. (Bottom) tSNE plots show the cell-type marker genes and AUCell scores for TEAD-TP63 direct induced/repressed target gene sets in and human lung scRNA-seq data from Travaglini *et al*. Only the epithelial cells are shown.
