# Supplementary material for: Convergence of YAP/TAZ, TEAD and TP63 activity is associated with bronchial premalignant severity and progression
Source: J Exp Clin Cancer Res. 2023 May 8;42:116. doi: 10.1186/s13046-023-02674-5 (PMC10165825; doi:10.1186/s13046-023-02674-5)
Supplement: Supplementary file 2 — Additional file 2: Supplementary Figure 1. TP63 isoform expression levels in TCGA-LUSC and in bronchial PML biopsy data related to Figure 1. Supplementary Figure 2. ChIP-seq analysis of YAP/TEAD/TP63 chromatin binding profiles related to Figure 2. Supplementary Figure 3. Transcriptomic analysis of TEAD-TP63 direct regulated target genes related to Figure 3. Supplementary Figure 4. Transcriptomic analysis of TEAD-TP63 direct regulated target genes in human bronchial PML data and lung scRNA-seq data related to Figure 4. Supplementary Figure 5. Analysis of CIITA in human bronchial PML data and lung scRNAseq data related to Figure 5. [file 13046_2023_2674_MOESM2_ESM.zip › Suppl5.pdf]

Supplementary Figure S5

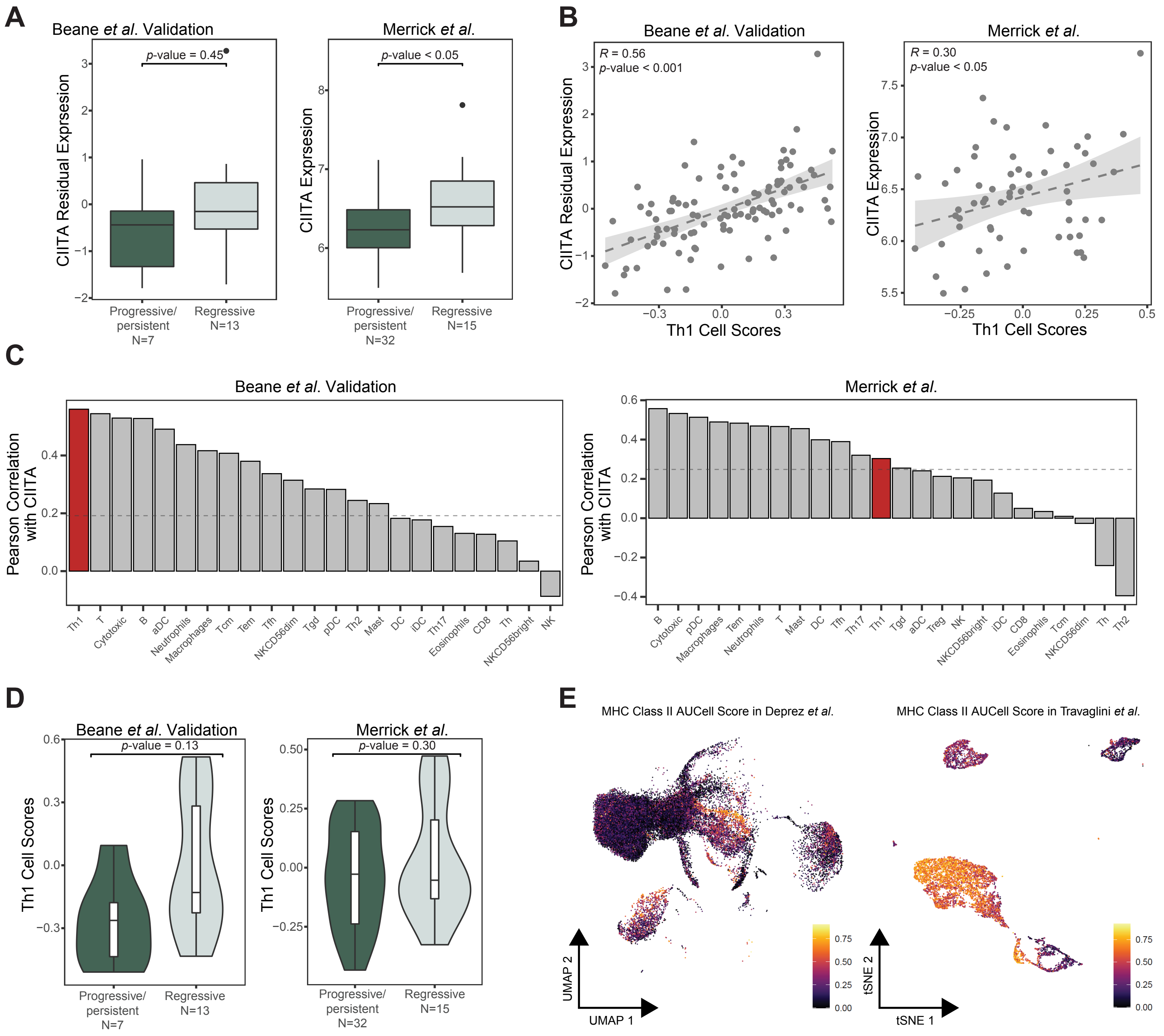

Supplementary Figure 5. Analysis of CIITA in human bronchial PML data and lung scRNA-seq data related to Figure 5.

a. (Left) Expression level of CIITA in progressive/persistent and regressive PML samples of the Proliferative subtype in Beane *et al*. Validation cohort. (Right) Expression level of CIITA in progressive/persistent and regressive PML samples in Merrick *et al*.

b. Scatter plots show the Pearson correlation between the expression level of CIITA and Th1 scores (calculated using GSVA based on genes from Bindea *et al*.) in Beane *et al*. Validation cohort (left) and Merrick *et al*. (right).

c. Immune cell-type ranked by their Pearson correlation coefficients with CIITA expression levels in Beane *et al*. Validation cohort (Left) and in Merrick *et al*. (Right). The dashed line indicates the Pearson correlation coefficient that reaches p-value = 0.05.

d. (Left) Th1 cell scores in progressive/persistent and regressive PML samples of the Proliferative subtype in Beane *et al*. Validation cohort. (Right) Th1 cell scores in progressive/persistent and regressive PML samples in Merrick *et al*.

e. (Left) UMAP plots show the MHC Class II gene (shown in Figure 5c) metagene scores calculated with AUCell in the healthy human airway scRNA-seq data from Deprez *et al*. (Right) tSNE plots show the MHC Class II gene (shown in Figure 5c) metagene scores calculated with AUCell in and human lung scRNA-seq data from Travaglini *et al*. Only the epithelial cells are shown.
